# Rounding the corner on residual risk: Implications of REDUCE‐IT for omega‐3 polyunsaturated fatty acids treatment in secondary prevention of atherosclerotic cardiovascular disease

**DOI:** 10.1002/clc.23220

**Published:** 2019-06-29

**Authors:** Seth J. Baum, Kenneth P. Scholz

**Affiliations:** ^1^ Excel Medical Clinical Trials Boca Raton Florida; ^2^ Department of Integrated Medical Science, Florida Atlantic University Charles E. Schmidt College of Medicine Boca Raton Florida; ^3^ Medical Writer Bend Oregon

**Keywords:** atherosclerotic cardiovascular disease, docosahexaenoic acid, eicosapentaenoic acid, icosapent ethyl

## Abstract

Patients with established atherosclerotic cardiovascular (CV) disease remain at increased risk of major adverse cardiovascular events even during optimal lipid‐lowering therapy. Recent studies using the methods of Mendelian randomization, as well as analyses of data from large statin trials, have concluded that elevated triglyceride (TG) levels contribute to that increased risk. Omega‐3 polyunsaturated fatty acids (omega‐3 PUFAs) from fish and shellfish (eicosapentaenoic acid [EPA] and docosahexaenoic acid [DHA]) reduce TG levels when added to the diet in sufficient amounts, and they have favorable effects on several other markers of CV risk. However, trials of omega‐3 PUFAs have had inconsistent findings regarding CV risk reduction. Recently, the REDUCE‐IT (Reduction of Cardiovascular Events with EPA‐Intervention Trial) trial reported that treatment of such high‐risk patients with icosapent ethyl, a purified and stabilized ethyl ester of EPA, reduced the risk of the trial's primary CV endpoint by 25% (95% confidence intervals [CI], 32%‐17%; *P* < .001). To appreciate the clinical implications of this result, it is important to understand how the REDUCE‐IT trial differed from previous trials, especially with regard to patient enrollment criteria and treatment dosing. We discuss these design features relative to other trials. TG lowering can account for only part of the risk reduction seen with icosapent ethyl; we also consider other potential contributory mechanisms.

## 1 INTRODUCTION

In November 2018, results of the REDUCE‐IT (Reduction of Cardiovascular Events with EPA‐Intervention Trial) trial were published.^1^ The trial demonstrated that treatment with a high dose (4 g per day) of the omega‐3 polyunsaturated fatty acid (PUFA) icosapent ethyl significantly reduced the risk of ischemic cardiovascular (CV) events and CV death in patients who (a) were on a stable dose of statin, (b) had established CV disease or type 2 diabetes mellitus and at least one additional CV risk factor, and (c) had elevated triglyceride (TG) levels at baseline (>135 or 150 mg/dL). These results were remarkable because they followed a series of major TG‐lowering trials that had failed to achieve reductions in CV outcomes in similar groups of patients.^2^, ^3^, ^4^, ^5^, ^6^ Why was REDUCE‐IT different? Are the results due solely to lowering of TG levels? What do the results mean for prevention of CV events in patients with established CV disease and elevated TG levels?

## 2 RESIDUAL RISK

In recent decades, statins have become the mainstay of lipid‐lowering therapy to reduce risk of CV events in patients with atherosclerotic cardiovascular disease (ASCVD).^5^ Statin therapy, which primarily targets low‐density lipoprotein cholesterol (LDL‐C), has a firmly established role in reducing the risk of CV events by 25% to 35% in such patients.^5^ The reduction in risk is related to the absolute reduction in LDL‐C levels.^7^ However, despite successful lowering of LDL‐C levels with statins, patients with ASCVD remain at elevated risk of CV events.^8^ PCSK9 (proprotein convertase subtilisin‐kexin type 9) inhibitors further reduce LDL‐C levels and CV event rates, but excess CV risk persists (eg, 3‐year event rates of approximately 10‐13 percent) even in patients with median LDL‐C levels of 30 to 40 mg/dL achieved with the combination of a statin and a PCSK9 inhibitor.^9^, ^10^ Although non‐lipid‐related factors—such as smoking, obesity, and diabetes mellitus—account for some of this residual risk,^8^ elevated TG levels or low high‐density lipoprotein cholesterol (HDL‐C) levels have been suspected, since they have been shown to be markers of residual risk in analyses of data from several large statin trials.^11^, ^12^, ^13^, ^14^, ^15^ Other lipid and lipoprotein fractions—such as non‐HDL‐C, apolipoprotein B, and LDL particle number—are also markers of residual risk.^16^

There is a strong inverse relationship between TG levels and HDL‐C levels; individuals with low HDL‐C levels generally have elevated TG levels and vice versa.^17^ However, because observational studies have consistently found a link between low HDL‐C and CV risk,^18^, ^19^, ^20^, ^21^, ^22^, ^23^ several clinical trials were conducted in an effort to reduce CV risk by administering treatments intended to increase HDL‐C levels.^24^, ^25^, ^26^, ^27^, ^28^ Although the treatments substantially increased HDL‐C levels, only one trial reported a small decrease in major CV event rates, and that decrease was consistent with the degree of LDL‐C lowering.^28^ We now have a better understanding of why efforts to increase HDL‐C levels have had such limited success in reducing CV event rates; recent studies using the method of Mendelian randomization (MR) have supported the conclusion that low HDL‐C is *not causally* associated with increased CV risk.^29^, ^30^, ^31^ These studies have revealed that genetic variants influencing HDL‐C levels do not significantly affect CV risk. Thus, although a low HDL‐C level is a marker of increased CV risk, there is now a strong body of evidence from both clinical trials and MR studies that a low HDL‐C level is *not causally* related to an increased risk of CV events.^32^

During the era just described, TG re‐emerged as an independent CV risk factor. As recently as 2011, a scientific statement from the American Heart Association concluded that the role of TG as an independent causal factor for CV disease was “debatable.”^17^ Subsequently, MR studies provided strong evidence that elevated TG‐rich lipoproteins (TRLs) *are causally* related to increased risk for CV events.^30^, ^32^, ^33^, ^34^, ^35^, ^36^ Furthermore, analyses of statin trials have found that patients with elevated TG levels after statin therapy are at particularly high risk for CV events. Thus, even though statins (as well as ezetimibe and PCSK9 inhibitors) reduce TG levels along with lowering LDL‐C,^37^ some patients continue to have elevated TG levels, which is now recognized as a risk factor for CV events. These developments have re‐invigorated research efforts to identify and develop strategies to reduce TG levels and TRLs.^38^

Several diet and lifestyle interventions—including weight loss, physical activity, moderation of alcohol consumption, and Mediterranean‐style diet—are recommended for reducing elevated TG levels.^17^ The mainstays of TG‐lowering pharmacotherapies had been fibrates and niacin, despite the fact that these agents had very little success in lowering CV risk. More recently, attention has turned to the omega‐3 fatty acids eicosapentaenoic acid (EPA) and docosahexaenoic acid (DHA), which are found in high concentrations in oily fishes and are known to reduce blood TG levels.^39^, ^40^ Indeed, low consumption of EPA and DHA has been associated with increased risk for CV disease in some observational studies.^40^ Beyond their effects on TG levels, omega‐3 PUFAs have numerous biological effects that may influence CV risk. Therefore, before discussing clinical trials of omega‐3 PUFAs for CV risk reduction, we will briefly review the biology of EPA and DHA.

### 2.1 EPA and DHA: TG lowering and beyond

Essential fatty acids are those fats that cannot be synthesized by humans; they must be consumed. The two essential fatty acids in the human diet are linoleic acid (LA; an omega‐6 FA) and alpha‐linolenic acid (ALA; an omega‐3 PUFA).^41^ LA is the precursor for arachidonic acid, and ALA is the precursor for EPA (and minimally DHA).^42^ The main dietary sources of ALA are plants and plant products (such as vegetable oils). In theory, humans possess enzymes necessary to synthesize EPA and DHA from plant‐based ALA. In reality, the production of EPA and DHA from ALA is inconsequential. Making matters worse, omega‐6 PUFAs, which are often overly abundant in the western diet, dampen the conversion of ALA to EPA and DHA.^42^, ^43^ Thus, many experts consider EPA and DHA to be essential fatty acids.^43^

Key dietary sources of EPA and DHA are oily fishes,^42^ and several epidemiologic studies have found that groups of people who consume large amounts of such fish have lower rates of CV events and CV death compared with other populations (reviewed in Nishizaki et al.^44^). Other studies, however, did not find a significant protective effect from consumption of a diet high in fish or EPA or DHA.^45^ These studies varied widely with regard to the amount and types of PUFAs consumed by the study population. In 2006, a pooled analysis of prospective studies and randomized trials concluded that consumption of fish or fish oil was associated with a 36% reduction in the relative risk of death from coronary heart disease.^46^ Thus, at that time, the prevailing medical opinion was that omega‐3 PUFAs from fish had a favorable effect on CV outcomes.

Further support for a role of EPA and DHA in CV risk reduction came from biomarker studies. A pooled analysis of cohort studies conducted around the world found that higher levels of plasma or phospholipid omega‐3 PUFAs (EPA, DHA, docosapentaenoic acid [DPA], and plant‐derived ALA) were associated with a modest (approximately 10%) but significantly lower risk of fatal CHD.^47^ When evaluating both observational and interventional studies of omega‐3 PUFAs, it is important, however, to consider whether the investigators have distinguished ALA, EPA, and, DHA. We now understand that there are substantial differences in their biological activities.^48^ Thus, studies that combine the omega‐3 PUFAs may fail to discern important health effects that are mostly due to one particular omega‐3 PUFA.

Studies have consistently found that fish‐oil consumption, as well as consumption of purified EPA or DHA, robustly reduce serum TG levels.^49^, ^50^, ^51^, ^52^, ^53^ ALA, in contrast, has only small and inconsistent effects on TG levels.^49^, ^50^ In most of the studies that found TG‐lowering effects of fish oil (or EPA/DHA), participants received 1 g to >4 g of the respective fatty acid per day.^52^ The TG lowering effect is linearly dose‐dependent, and individuals with higher baseline TG levels exhibit greater declines in absolute TG levels in response to omega‐3 PUFA consumption.^40^

**Figure 1:**
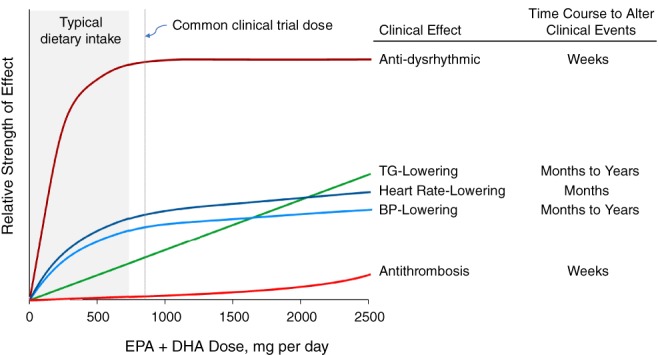
Conceptual depiction of the effects of fish‐derived omega‐3 polyunsaturated fatty acids on cardiovascular risk factors, including estimated dose dependence. Dose dependence was estimated from analyses of multiple studies of each risk factor. Modified from Mozaffarian and Rimm,^46^ used with permission

Reported biological actions of EPA and DHA extend well beyond TG lowering and include effects expected to be cardioprotective.^48^, ^54^, ^55^, ^56^, ^57^ As summarized by Mozaffarian and Rimm,^46^ and shown in Figure 1, fish oil omega‐3 PUFAs have been linked with reduced blood pressure and heart rate, as well as decreased platelet aggregation.^40^, ^58^, ^59^, ^60^, ^61^ They have also been linked with anti‐inflammatory actions and stabilization of coronary plaque.^62^, ^63^, ^64^, ^65^ Anti‐dysrhythmic actions have been observed in animal or cell models, but their significance in humans is controversial.^40^ As shown in Figure 1, some of the observed cardioprotective effects of omega‐3 PUFAs require comparatively high doses of EPA/DHA; for example, lowering of diastolic blood pressure seems to require doses of ≥2 g per day,^59^ and antithrombotic effects appear to require even higher doses.^66^ It is plausible that other biological effects of omega‐3 PUFAs—such as contributions to the mediation of inflammation^67^, ^68^—may contribute to improvements in CV endpoints, although those relationships need extensive additional testing.

## 3 RANDOMIZED CONTROLLED TRIALS AND META‐ANALYSES

Large‐scale, randomized trials have examined three classes of treatments targeting TG levels: fibrates, niacin, and omega‐3 PUFAs. These trials have been thoroughly discussed by other recent reviews,^5^, ^17^, ^44^, ^69^ so we will briefly summarize the state of the field up to 2018. Then we will discuss very recent studies, including three trials of omega‐3 PUFAs that were published in 2018.

### 3.1 Fibrates and niacin

As noted by prior reviews,^5^, ^70^ trials of fibrates or niacin added to statin therapy have failed to show a benefit of the added treatment in terms of CV event rates.^12^, ^25^, ^26^, ^71^, ^72^ The ongoing PROMINENT trial, involving the novel fibrate pemafibrate,^73^ may provide insight into this question (NCT03071692).

### 3.2 Fish oil omega‐3 PUFAs: Background of clinical studies

A 2011 review analyzed randomized controlled trials, prospective cohort studies, and meta‐analyses conducted up to that time and concluded that consumption of fish or fish oil reduced coronary heart disease mortality in populations with and without established CV disease.^40^ Three meta‐analyses of randomized controlled trials of omega‐3 PUFA supplements supported this conclusion.^46^, ^74^, ^75^ However, another meta‐analysis of randomized controlled trials published in 2012 concluded that omega‐3 PUFA supplementation was not associated with significant reduction in the risk of all‐cause death, cardiac death, or myocardial infarction.^76^ That meta‐analysis, however, used an unusually high threshold for statistical significance (*α* = 0.0063). It is remarkable, for example, that all 17 of the trials included in the analysis of all‐cause death reported a relative risk <0.96 favoring omega‐3 PUFA, and seven of those trials reported upper confidence intervals that were < 1.0, yet the meta‐analysis failed to find a significant benefit of omega‐3 PUFAs for all‐cause death. The authors noted that there was extensive heterogeneity across trials with regard to event rates, baseline CV disease risk, treatment setting (eg, primary prevention vs secondary prevention), co‐administered therapies, baseline intake of omega‐3 PUFAs, dose, and nature of omega‐3 PUFA used for the intervention, and treatment adherence. Thus, heterogeneity was likely so high that a meaningful conclusion could not be reached. A more recent meta‐analysis revealed the importance of such heterogeneity. That study^77^ also found a non‐significant reduction in coronary heart disease risk across the entire study group (summary relative risk estimate [SSRE], 0.94; 95% CI 0.85‐1.05), but it found that high‐risk populations obtained significant benefits from omega‐3 PUFA interventions. Specifically, patients with elevated baseline TG levels had an SSRE of 0.86 (95% CI 0.76‐0.98). The authors also found evidence that higher intakes of omega‐3 PUFAs were associated with significantly greater reductions in coronary heart disease risk (SSRE, 0.82; 95% CI 0.74‐0.92). These meta‐analyses reinforce the importance of trial design; patient subgroups should be carefully and prospectively defined and studied in clinical trials, and doses and compositions of omega‐3 PUFAs need to be precisely constructed and controlled.

### 3.3 Fish oil omega‐3 PUFAs: Recent trials

In 2018, the results of two major trials (ASCEND and VITAL) of omega‐3 PUFA (1 g per day) for *primary* prevention of CV disease were reported.^4^, ^78^ Neither trial reported a significant reduction in the risk of a composite cardiovascular endpoint in the omega‐3 PUFA group compared to the placebo group, although the VITAL trial reported a significantly lower rate of myocardial infarction in the omega‐3 PUFA arm. Both trials excluded patients with established CV disease, and neither trial required statin therapy or enrolled patients on the basis of elevated TG levels. As discussed in more detail below, both trials may have used inadequate doses of omega‐3 PUFAs. Another possibility is that the trial population was at too low risk to reveal statistically significant benefits of omega‐3 PUFAs. A third major trial reported in 2018 (REDUCE‐IT) studied an omega‐3 PUFA in patients with established CV disease or diabetes mellitus and at least one additional CV risk factor.^1^ In addition, participants were required to be on a stable dose of statin and to have an elevated fasting TG level (150‐499 mg/dL). The trial enrolled 8179 patients (71% of whom had established CV disease) and randomized them to icosapent ethyl (a purified and stable ethyl ester of EPA; 2 g twice daily) or placebo. The primary endpoint was a composite of cardiovascular death, MI, stroke, coronary revascularization, or unstable angina. After a median follow‐up of 4.9 years, the icosapent ethyl group had a significantly lower rate of primary endpoint events (17.2% of patients) compared to the placebo group (22.0%; HR, 0.75; 95% CI, 0.68‐0.83; *P* = .001). A similar benefit of icosapent ethyl was found for the secondary endpoint of CV death, MI, and stroke (HR, 0.74; 95% CI, 0.65‐0.83; *P* < .001). The benefits of icosapent ethyl were observed across a broad set of patient subgroups based on baseline characteristics, including TG and LDL‐C levels and the presence or absence of diabetes mellitus. It is important to note that all study participants had elevated TG levels at baseline (most >150 mg/dL).

In the ASCEND and VITAL trials, there were no significant differences between active treatment groups and the placebo groups with regard to rates of non‐fatal serious adverse events, including bleeding.^4^, ^78^ In REDUCE‐IT, icosapent ethyl was associated with a small but significant increase in rates of hospitalization for atrial fibrillation or flutter vs placebo (3.1% vs 2.1%; *P* = .004) and a trend toward increased rates of serious bleeding (2.7% vs 2.1%; *P* = .06).^1^ However, prior reviews of bleeding risk associated with pharmacotherapeutic use of omega‐3 PUFAs (at daily doses of 1‐6 g) concluded that there was no effect on the risk of clinically significant bleeding.^79^, ^80^

## 4 WAS REDUCE‐IT DIFFERENT?

At first glance, it may seem that the results of REDUCE‐IT are at odds with those from prior trials of omega‐3 PUFAs. On closer inspection, it becomes evident that the results of REDUCE‐IT are consistent with observations from several previous studies. As already discussed, the most recent meta‐analysis of omega‐3 PUFA supplements for reducing the risk of CV events found that high‐risk patients, especially those with elevated TG levels, were more likely to obtain benefit from omega‐3 PUFA treatment.^77^ Similarly, a previous open‐label trial in Japan (JELIS, Japan EPA Lipid Intervention Study) found that icosapent ethyl (1.8 g per day) significantly reduced the risk of major coronary events in hypercholesterolemic patients (HR, 0.81; 95% CI 0.69‐0.95). Notably, patients in JELIS with elevated TG (≥150 mg/dL) and low HDL‐C levels (<40 mg/dL) had the highest risk of coronary events and the greatest reduction in event rates associated with icosapent ethyl treatment (HR, 0.47; 95% CI, 0.23‐0.98; *P* = .043). Similar results were found in subgroup analyses of REDUCE‐IT: patients who had the combination of baseline TG ≥150 mg/dL and baseline HDL‐C ≤ 35 mg/dL exhibited a HR for the primary endpoint of 0.62 (icosapant ethyl group vs placebo group; 95% CI, 0.51‐0.77). The corresponding HR for patients who did not meet those baseline criteria was 0.79 (95% CI, 0.71‐0.88; *P* = .04 for subgroup‐by‐treatment interaction). Independent confirmation of the importance of those baseline criteria may be needed to fully understand their clinical implications.

Table 1 presents a summary of the characteristics of major CV prevention trials of omega‐3 PUFAs, including the latest, REDUCE‐IT. Of these nine trials, only three—GISSI‐P, JELIS, and REDUCE‐IT—demonstrated significantly lower rates of the primary endpoint among patients in the omega‐3 PUFA group vs placebo. Notably, median baseline TG levels among participants of REDUCE‐IT (216 mg/dL) were substantially higher than in all other omega‐3 PUFA trials, with the other two positive trials having the second‐ and third‐highest baseline TG levels. REDUCE‐IT was the only omega‐3 PUFA trial that specifically required elevated TG levels at baseline. This prerequisite may be an important aspect of the trial's success. Observations from these studies suggest that elevated TG levels may be a marker for secondary CV prevention patients who are most likely to benefit from omega‐3 PUFA treatment.

**Table 1:** Summary characteristics and results of major cardiovascular prevention trials of omega‐3 PUFAs

	GISSI‐P^80^	JELIS^81^	GISSI‐HF^82^	OMEGA^83^	Alpha‐Omega^2^	SU.FOL.OM3^84^	ORIGIN^3^	Risk and Prevention^85^	VITAL^73^	ASCEND^4^	REDUCE‐IT^1^
Publication date	1999	2007	2008	2010	2010	2010	2012	2013	2018	2018	2018
N	11 324	18 645	6975	3851	4837	2501	12 536	12 513	25 871	15 480	8179
Median follow‐up, years	3.5	4.6	3.9	1 (treatment duration)	3.3	4.7	6.2	5	5.3	7.4	4.9
Enrollment Criteria											
Primary or secondary prevention	Secondary	Primary	Secondary	Secondary	Secondary	Secondary	Primary	Mixed	Primary	Primary	Mixed
Enrollment criteria	Recent MI (≤3 months)	Total‐C ≥ 251 mg/dL	Chronic HF, NYHA classes II‐IV	Recent MI (≤14 days)	Prior MI	History of MI, UA, or ischemic stroke	High risk + impaired fasting glucose, impaired glucose tolerance, or diabetes	ASCVD but no MI, or multiple risk factors	Men ≥50 years; Women ≥55 years	Diabetes mellitus, no evidence of CV disease	• Established CV disease (at least 70% of participants) • DM + other risk factors
Baseline TG levels, mg/dL	No enrollment requirement										135‐499
Baseline LDL, mg/dL	No requirement	≥170	No enrollment requirement								41‐100
Baseline statin required	No										Yes
Baseline lipid characteristics											
Median TG mg/dL	162‐163	153‐154	126	NR	144‐150	97‐115	140‐142	150	NR	NR	216
Mean or median, HDL‐C, mg/dL	41	58‐59	NR	NR	~50	43	46	51	NR	49	40
Mean or median, LDL‐C mg/dL	137‐138	182	NR	~49% had “hypercholesterolemia”	98‐102	2.6‐2.7	112	132‐133	NR	112‐113 (non‐HDL‐C)	75
Lipid lowering therapy, %	~5	100%, added to both arms	22‐23		86	83‐87	53‐55	41	38	75‐76	100
Treatment											
Active treatment (daily dose)	850 mg, EPA + DHA	1800 mg Icosapent ethyl	850 mg, EPA + DHA	460 mg EPA + 380 mg DHA	Margarine containing ALA, EPA, and DHA (Avg 376 mg, EPA + DHA per day)	600 mg, EPA + DHA (2:1)	465 mg EPA 375 mg DHA	850 mg, EPA + DHA	460 mg EPA + 380 mg DHA (+/− vitamin D)	460 mg EPA + 380 mg DHA	4 g Icosapent ethyl
Primary endpoint											
Primary endpoint	Death, MI, or stroke	Cardiac death, MI, UA, PCI, CABG	Co‐primary: Death, and Death or CV hospitalization	Sudden cardiac death	CV event (fatal or nonfatal), PCI, or CABG	CV death, MI, stroke	CV death	CV death or CV hospitalization	MI, stroke or CV death	First MI, stroke, TIA, or vascular death (excluding intracranial hemorrhage)	CV death, MI, stroke, coronary revascularization, UA
Result, HR (95% CI)	0.85 (0.74‐0.98) 4‐way analysis	0.81 (0.69–0.95)	Death: 0.91 (0.833‐0.998), death or CV Hosp: 0.92 (0.849‐0.999)	0.95 (0.56‐1.60)	1.01 (0.87‐1.17)	1.08 (0.79‐1.47)	0.98 (0.87‐1.10)	0.97 (0.88‐1.08)		0.97 (0.87‐1.08)	0.75(0.68–0.83)

Abbreviations: ALA, alpha‐linolenic acid; CABG, coronary artery bypass graft; CI, confidence interval; CV, cardiovascular; DHA, docosahexaenoic acid; EPA, eicosapentaenoic acid; HF, heart failure; HR, hazard ratio; MI, myocardial infarction; NR, not reported; PCI, percutaneous coronary intervention; UA, unstable angina.

Source: Modified and updated from Ganda et al.^5^ and Bhatt et al..^65^

Another salient distinction between REDUCE‐IT and prior trials was the dose of omega‐3 PUFA administered during the treatment period (4 g per day vs ≤850 mg per day in other large trials other than JELIS). The efficacy of high‐dose omega‐3 PUFA in REDUCE‐IT is understandable in the context of JELIS and a recent meta‐analysis, which found that higher intakes of omega‐3 PUFAs were associated with greater risk reductions.^77^ Median plasma levels of EPA among participants in the REDUCE‐IT trial in the icosapent ethyl arm were 26.1 μg/mL at baseline and 144 μg/mL after 1 year. In JELIS, EPA levels were substantially higher at baseline (93‐97 μg/mL) and increased to approximately 165 /mL during icosapent ethyl treatment.^81^ Furthermore, in JELIS, the hazard ratio for CV events was inversely related to plasma EPA concentrations, with notable declines in hazard ratios observed when plasma EPA concentrations exceeded 100‐150 μg/mL. Unfortunately, very few past trials of omega‐3 PUFAs have measured baseline or on‐treatment plasma EPA levels. It is important to recognize that the baseline levels reported in REDUCE‐IT are more likely to represent a typical Western population than the baseline levels reported in JELIS, because the Japanese population has a much higher average dietary intake of oily fish compared to populations consuming a typical western diet. Together, these considerations suggest that Western patients may require higher doses of EPA, such as those used in the REDUCE‐IT trial, to achieve meaningful reductions in CV event rates.

As already discussed, some of the beneficial effects of omega‐3 PUFAs, including their TG‐lowering effects, require comparatively high doses. Thus, it is likely that patients in the icosapent ethyl arm of REDUCE‐IT experienced a greater absolute reduction in TG levels during treatment as compared to other omega‐3 PUFA trials. However, the trial investigators reported that reduction in the primary endpoint or key secondary endpoint in REDUCE‐IT did not depend on whether patients achieved TG levels below or above 150 mg/dL at 1 year.^1^ This finding provides further evidence that biological effects beyond TG lowering contributed to the clinical benefits of icosapent ethyl in the REDUCE‐IT trial. More detailed analyses and perhaps additional clinical studies may be required to establish the dose‐dependence and biological mechanisms by which icosapent ethyl reduced the rates of major CV events.

Finally, it must be noted that icosapent ethyl is a pure, esterified form of EPA, and it was used in two of the three successful trials summarized in Table 1 (JELIS and REDUCE‐IT). Most other trials used formulations combining EPA and DHA in various concentrations. It is unknown to what degree these differences in drug formulation account for some of the inconsistencies in CV outcomes across trials. Although EPA and DHA share many biological effects, some differences have been reported.^54^, ^55^ The ongoing STRENGTH trial (NCT02104817), which uses a combination of EPA and DHA, may help to clarify the roles of omega‐3 PUFAs for secondary prevention of CV events.^82^

## 5 MECHANISM OF CLINICAL BENEFIT IN REDUCE‐IT

With several lines of evidence suggesting that elevated TG levels are a marker of the potential for secondary prevention patients to benefit from omega‐3 PUFAs, and with the now‐established role of elevated TG levels in increased CV risk, it is tempting to assume that the TG‐lowering effect of icosapent ethyl explains its CV benefits. Indeed, icosapent ethyl is already approved in the United States for reducing TG levels in patients with very high TG levels (≥500 mg/dL).^83^, ^84^ REDUCE‐IT, however, examined people with minimum baseline TG levels as low as 135 to 150 mg/dL. And it revealed evidence that TG lowering may not fully explain CV benefits of icosapent ethyl.^1^ First, the hazard ratio for the primary endpoint associated with icosapent ethyl vs placebo was similar in subgroups defined by baseline TG levels, regardless of whether the cutoffs were 150 or 200 mg/dL. Second, the benefits of treatment were not influenced by whether patients achieved TG levels <150 mg/dL during treatment.^1^ Although more detailed analyses are needed, the currently available analyses support the conclusion that TG lowering is not the sole mechanism responsible for lowering CV risk among the patients who participated in REDUCE‐IT. A similar conclusion was found in a post hoc analysis of the JELIS trial.^85^

Previous trials of icosapent ethyl in patients with high TG levels (≥200 mg/dL) found that treatment led to improvements in levels of several atherogenic lipid particles and biomarkers, including apolipoprotein B, very low density lipoprotein cholesterol, lipoprotein‐associated phospholipase A2, remnant‐like particle cholesterol, and apolipoprotein C‐III.^53^, ^86^, ^87^ Furthermore, preclinical studies have described several other beneficial effects of omega‐3 PUFAs that could translate into reductions in rates of clinical events—effects such as improving endothelial function and nitric oxide availability, reducing inflammatory cytokines and enzymes, reducing activation of platelets, and contributing to plaque stabilization (summarized in Ganda et al.^5^).

## 6 CONCLUSION

The REDUCE‐IT trial found that icosapent ethyl (4 g per day), a pure and stable form of the omega‐3 PUFA EPA, significantly reduced residual risk of cardiovascular events in patients with ASCVD and elevated TG levels (≥150 mg/dL) who were receiving a stable dose of statin at study entry. On superficial examination, the REDUCE‐IT trial appears to be an outlier among trials of omega‐3 PUFAs for secondary prevention of CV events. On closer inspection, however, its design and outcomes are consistent with lessons learned from previous studies. Baseline TG levels appear to be a marker of increased likelihood of benefiting from icosapent ethyl treatment, and treatment significantly lowers TG levels. However, currently available analyses suggest that reduction in TG levels likely do not fully account for the beneficial effects of treatment. Despite persistent questions regarding its full mechanism of action, icosapent ethyl significantly ameliorates residual CV risk in patients with ASCVD receiving statin therapy and has been associated with minimal adverse effects. It appears, therefore, that with the results of the REDUCE‐IT trial, we have rounded the corner; we now have in our sights the ability to further reduce the risk of CV events in appropriately selected patients with residual CV risk on optimal statin therapy.

## CONFLICT OF INTEREST

Seth J. Baum: Scientific Advisory Board: Sanofi, Amgen, Regeneron, Akcea; Consultant: Sanofi, Amgen, Cleveland Heart Labs, GLG Group, Guidepoint Global, Regeneron, Novo Nordisk, Akcea; Speaker: Amgen, Aralez, Boehringer‐Ingelheim, Novo Nordisk, Akcea. Kenneth P. Scholz was paid an hourly fee for preparation of the manuscript.

## References

[1] Bhatt DL , Steg PG , Miller M , et al. Cardiovascular risk reduction with Icosapent ethyl for hypertriglyceridemia. N Engl J Med. 2018;380:11‐22. 30415628 10.1056/NEJMoa1812792

[2] Kromhout D , Giltay EJ , Geleijnse JM , Alpha Omega Trial Group . N‐3 fatty acids and cardiovascular events after myocardial infarction. N Engl J Med. 2010;363(21):2015‐2026. 20929341 10.1056/NEJMoa1003603

[3] The Origin Trial Investigators . N‐3 fatty acids and cardiovascular outcomes in patients with dysglycemia. N Engl J Med. 2012;367(4):309‐318. 22686415 10.1056/NEJMoa1203859

[4] The Ascend Study Collaborative Group . Effects of n‐3 fatty acid supplements in diabetes mellitus. N Engl J Med. 2018;379(16):1540‐1550. 30146932 10.1056/NEJMoa1804989

[5] Ganda OP , Bhatt DL , Mason RP , Miller M , Boden WE . Unmet need for adjunctive dyslipidemia therapy in hypertriglyceridemia management. J Am Coll Cardiol. 2018;72(3):330‐343. 29935936 10.1016/j.jacc.2018.04.061

[6] Aung T , Halsey J , Kromhout D , et al. Associations of Omega‐3 fatty acid supplement use with cardiovascular disease risks: meta‐analysis of 10 trials involving 77917 individuals. JAMA Cardiol. 2018;3(3):225‐234. 29387889 10.1001/jamacardio.2017.5205 PMC5885893

[7] Silverman MG , Ference BA , Im K , et al. Association between lowering LDL‐C and cardiovascular risk reduction among different therapeutic interventions: a systematic review and meta‐analysis. JAMA. 2016;316(12):1289‐1297. 27673306 10.1001/jama.2016.13985

[8] Mora S , Wenger NK , Demicco DA , et al. Determinants of residual risk in secondary prevention patients treated with high‐ versus low‐dose statin therapy: the treating to new targets (TNT) study. Circulation. 2012;125(16):1979‐1987. 22461416 10.1161/CIRCULATIONAHA.111.088591 PMC3338158

[9] Sabatine MS , Giugliano RP , Keech AC , et al. Evolocumab and clinical outcomes in patients with cardiovascular disease. N Engl J Med. 2017;376(18):1713‐1722. 28304224 10.1056/NEJMoa1615664

[10] Schwartz GG , Steg PG , Szarek M , et al. Alirocumab and cardiovascular outcomes after acute coronary syndrome. N Engl J Med. 2018;379(22):2097‐2107. 30403574 10.1056/NEJMoa1801174

[11] Miller M , Cannon CP , Murphy SA , Qin J , Ray KK , Braunwald E . Impact of triglyceride levels beyond low‐density lipoprotein cholesterol after acute coronary syndrome in the PROVE IT‐TIMI 22 trial. J Am Coll Cardiol. 2008;51(7):724‐730. 18279736 10.1016/j.jacc.2007.10.038

[12] Ginsberg HN , Elam MB , Lovato LC , et al. Effects of combination lipid therapy in type 2 diabetes mellitus. N Engl J Med. 2010;362(17):1563‐1574. 20228404 10.1056/NEJMoa1001282 PMC2879499

[13] Cholesterol Treatment Trialists' (CTT) Collaborators , Kearney PM , Blackwell L , Collins R , et al. Efficacy of cholesterol‐lowering therapy in 18,686 people with diabetes in 14 randomised trials of statins: a meta‐analysis. Lancet. 2008;371(9607):117‐125. 18191683 10.1016/S0140-6736(08)60104-X

[14] Faergeman O , Holme I , Fayyad R , et al. Plasma triglycerides and cardiovascular events in the treating to new targets and incremental decrease in end‐points through aggressive lipid lowering trials of statins in patients with coronary artery disease. Am J Cardiol. 2009;104(4):459‐463. 19660594 10.1016/j.amjcard.2009.04.008

[15] Schwartz GG , Abt M , Bao W , et al. Fasting triglycerides predict recurrent ischemic events in patients with acute coronary syndrome treated with statins. J Am Coll Cardiol. 2015;65(21):2267‐2275. 26022813 10.1016/j.jacc.2015.03.544

[16] Mora S , Glynn RJ , Boekholdt SM , Nordestgaard BG , Kastelein JJ , Ridker PM . On‐treatment non‐high‐density lipoprotein cholesterol, apolipoprotein B, triglycerides, and lipid ratios in relation to residual vascular risk after treatment with potent statin therapy: JUPITER (justification for the use of statins in prevention: an intervention trial evaluating rosuvastatin). J am Coll Cardiol. 2012;59(17):1521‐1528. 22516441 10.1016/j.jacc.2011.12.035 PMC3338194

[17] Miller M , Stone NJ , Ballantyne C , et al. Triglycerides and cardiovascular disease: a scientific statement from the American Heart Association. Circulation. 2011;123(20):2292‐2333. 21502576 10.1161/CIR.0b013e3182160726

[18] Kannel WB , Dawber TR , Friedman GD , et al. Risk factors in coronary heart disease. An evaluation of several serum lipids as predictors of coronary heart disease; the Framingham study. Ann Intern Med. 1964;61:888‐899. 14233810 10.7326/0003-4819-61-5-888

[19] Castelli WP , Anderson K , Wilson PWF , Levy D . Lipids and risk of coronary heart disease. The Framingham Study. Ann Epidemiol. 1992;2(1–2):23‐28. 1342260 10.1016/1047-2797(92)90033-m

[20] Wilson PW , Abbott RD , Castelli WP . High density lipoprotein cholesterol and mortality. The Framingham Heart Study. Arteriosclerosis. 1988;8(6):737‐741. 3196218 10.1161/01.atv.8.6.737

[21] Di Angelantonio E , Sarwar N , Perry P , et al. Major lipids, apolipoproteins, and risk of vascular disease. JAMA. 2009;302(18):1993‐2000. 19903920 10.1001/jama.2009.1619 PMC3284229

[22] Lewington S , Whitlock G , Clarke R , et al. Blood cholesterol and vascular mortality by age, sex, and blood pressure: a meta‐analysis of individual data from 61 prospective studies with 55,000 vascular deaths. Lancet. 2007;370(9602):1829‐1839. 18061058 10.1016/S0140-6736(07)61778-4

[23] Acharjee S , Boden WE , Hartigan PM , et al. Low levels of high‐density lipoprotein cholesterol and increased risk of cardiovascular events in stable ischemic heart disease patients: a post‐hoc analysis from the COURAGE trial (clinical outcomes utilizing revascularization and aggressive drug evaluation). J Am Coll Cardiol. 2013;62(20):1826‐1833. 23973693 10.1016/j.jacc.2013.07.051 PMC5661970

[24] Barter PJ , Caulfield M , Eriksson M , et al. Effects of torcetrapib in patients at high risk for coronary events. N Engl J Med. 2007;357(21):2109‐2122. 17984165 10.1056/NEJMoa0706628

[25] Boden WE , Probstfield JL , Anderson T , et al. Niacin in patients with low HDL cholesterol levels receiving intensive statin therapy. N Engl J Med. 2011;365(24):2255‐2267. 22085343 10.1056/NEJMoa1107579

[26] Landray MJ , Haynes R , Hopewell JC , et al. Effects of extended‐release niacin with laropiprant in high‐risk patients. N Engl J Med. 2014;371(3):203‐212. 25014686 10.1056/NEJMoa1300955

[27] Schwartz GG , Olsson AG , Abt M , et al. Effects of dalcetrapib in patients with a recent acute coronary syndrome. N Engl J Med. 2012;367(22):2089‐2099. 23126252 10.1056/NEJMoa1206797

[28] The HPS3/TIMI55‐REVEAL Collaborative Group , Bowman L , Hopewell JC , et al. Effects of anacetrapib in patients with atherosclerotic vascular disease. N Engl J Med. 2017;377(13):1217‐1227. 28847206 10.1056/NEJMoa1706444

[29] Burgess S , Harshfield E . Mendelian randomization to assess causal effects of blood lipids on coronary heart disease: lessons from the past and applications to the future. Curr Opin Endocrinol Diabetes Obes. 2016;23(2):124‐130. 26910273 10.1097/MED.0000000000000230 PMC4816855

[30] Holmes MV , Asselbergs FW , Palmer TM , et al. Mendelian randomization of blood lipids for coronary heart disease. Eur Heart J. 2015;36(9):539‐550. 24474739 10.1093/eurheartj/eht571 PMC4344957

[31] Voight BF , Peloso GM , Orho‐Melander M , et al. Plasma HDL cholesterol and risk of myocardial infarction: a mendelian randomisation study. Lancet. 2012;380(9841):572‐580. 22607825 10.1016/S0140-6736(12)60312-2 PMC3419820

[32] Musunuru K , Kathiresan S . Surprises from genetic analyses of lipid risk factors for atherosclerosis. Circ Res. 2016;118(4):579‐585. 26892959 10.1161/CIRCRESAHA.115.306398 PMC4762058

[33] Crosby J , Peloso GM , Auer PL , et al. Loss‐of‐function mutations in APOC3, triglycerides, and coronary disease. N Engl J Med. 2014;371(1):22‐31. 24941081 10.1056/NEJMoa1307095 PMC4180269

[34] Jorgensen AB , Frikke‐Schmidt R , Nordestgaard BG , et al. Loss‐of‐function mutations in APOC3 and risk of ischemic vascular disease. N Engl J Med. 2014;371(1):32‐41. 24941082 10.1056/NEJMoa1308027

[35] Jorgensen AB , Frikke‐Schmidt R , West AS , et al. Genetically elevated non‐fasting triglycerides and calculated remnant cholesterol as causal risk factors for myocardial infarction. Eur Heart J. 2013;34(24):1826‐1833. 23248205 10.1093/eurheartj/ehs431

[36] Varbo A , Benn M , Tybjaerg‐Hansen A , et al. Remnant cholesterol as a causal risk factor for ischemic heart disease. J Am Coll Cardiol. 2013;61(4):427‐436. 23265341 10.1016/j.jacc.2012.08.1026

[37] Fiengold KR , Grunfeld C . Cholesterol Lowering Drugs In: Feingold KR, Anawalt B, Boyce A, et al., eds. Endotext. South Dartmouth, MA: MDText.com, Inc.; 2018.

[38] Koh KK . How to control residual risk during the statin era? J Am Coll Cardiol. 2015;66(16):1848. 26483117 10.1016/j.jacc.2015.07.072

[39] Harris WS , Bulchandani D . Why do omega‐3 fatty acids lower serum triglycerides? Curr Opin Lipidol. 2006;17(4):387‐393. 16832161 10.1097/01.mol.0000236363.63840.16

[40] Mozaffarian D , Wu JHY . Omega‐3 fatty acids and cardiovascular disease: effects on risk factors, molecular pathways, and clinical events. J Am Coll Cardiol. 2011;58(20):2047‐2067. 22051327 10.1016/j.jacc.2011.06.063

[41] Food and Agriculture Organization of the United Nation . Fats and Fatty Acids in Human Nutrition: Report of an Expert Consultation. Rome, Italy: Food and Agriculture Organization of the United Nations; 2016.

[42] Calder PC , Yaqoob P . Understanding omega‐3 polyunsaturated fatty acids. Postgrad Med. 2009;121(6):148‐157. 10.3810/pgm.2009.11.2083 19940425

[43] Cholewski M , Tomczykowa M , Tomczyk M . A comprehensive review of chemistry, sources and bioavailability of omega‐3 fatty acids. Nutrients. 2018;10(11):1662. 10.3390/nu10111662 PMC6267444 30400360

[44] Nishizaki Y , Shimada K , Daida H . The balance of omega‐3 polyunsaturated fatty acids for ‐reducing residual risks in patients with coronary artery disease. Acta Cardiol. 2017;72(3):240‐248. 28636510 10.1080/00015385.2017.1305174

[45] Yamagishi K , Iso H , Date C , et al. Fish, omega‐3 polyunsaturated fatty acids, and mortality from cardiovascular diseases in a nationwide community‐based cohort of Japanese men and women the JACC (Japan collaborative cohort study for evaluation of cancer risk) study. J Am Coll Cardiol. 2008;52(12):988‐996. 18786479 10.1016/j.jacc.2008.06.018

[46] Mozaffarian D , Rimm EB . Fish intake, contaminants, and human health: evaluating the risks and the benefits. JAMA. 2006;296(15):1885‐1899. 17047219 10.1001/jama.296.15.1885

[47] Del Gobbo LC , Imamura F , Aslibekyan S , et al. Omega‐3 polyunsaturated fatty acid biomarkers and coronary heart disease: pooling project of 19 cohort studies. JAMA Intern Med. 2016;176(8):1155‐1166. 27357102 10.1001/jamainternmed.2016.2925 PMC5183535

[48] Innes JK , Calder PC . The differential effects of eicosapentaenoic acid and docosahexaenoic acid on cardiometabolic risk factors: a systematic review. Int J Mol Sci. 2018;19(2):532. 10.3390/ijms19020532 PMC5855754 29425187

[49] Harris WS . n‐3 fatty acids and serum lipoproteins: human studies. Am J Clin Nutr. 1997;65(5 Suppl):1645s‐1654s. 9129504 10.1093/ajcn/65.5.1645S

[50] Balk EM , Lichtenstein AH , Chung M , Kupelnick B , Chew P , Lau J . Effects of omega‐3 fatty acids on serum markers of cardiovascular disease risk: a systematic review. Atherosclerosis. 2006;189(1):19‐30. 16530201 10.1016/j.atherosclerosis.2006.02.012

[51] Eslick GD , Howe PR , Smith C , et al. Benefits of fish oil supplementation in hyperlipidemia: a systematic review and meta‐analysis. Int J Cardiol. 2009;136(1):4‐16. 18774613 10.1016/j.ijcard.2008.03.092

[52] Leslie MA , Cohen DJ , Liddle DM , et al. A review of the effect of omega‐3 polyunsaturated fatty acids on blood triacylglycerol levels in normolipidemic and borderline hyperlipidemic individuals. Lipids Health Dis. 2015;14:53. 26048287 10.1186/s12944-015-0049-7 PMC4488064

[53] Brinton EA , Ballantyne CM , Bays HE , Kastelein JJ , Braeckman RA , Soni PN . Effects of icosapent ethyl on lipid and inflammatory parameters in patients with diabetes mellitus‐2, residual elevated triglycerides (200‐500 mg/dL), and on statin therapy at LDL‐C goal: the ANCHOR study. Cardiovasc Diabetol. 2013;12:100. 23835245 10.1186/1475-2840-12-100 PMC3718763

[54] Baum SJ , Hamm A . Fatty acids and their derivatives in cardiovascular disease: Arachidonic, eicosapentaenoic, and docosahexaenoic acids and their byproducts, the eicosanoids and docosanoids. Curr Cardiovasc Risk Rep. 2012;6:146‐154.

[55] Bird JK , Calder PC , Eggersdorfer M . The role of n‐3 long chain polyunsaturated fatty acids in cardiovascular disease prevention, and interactions with statins. Nutrients. 2018;10(6):775. 10.3390/nu10060775 PMC6024670 29914111

[56] Nelson JR , True WS , Le V , et al. Can pleiotropic effects of eicosapentaenoic acid (EPA) impact residual cardiovascular risk? Postgrad Med. 2017;129(8):822‐827. 28974128 10.1080/00325481.2017.1385365

[57] Borow KM , Nelson JR , Mason RP . Biologic plausibility, cellular effects, and molecular mechanisms of eicosapentaenoic acid (EPA) in atherosclerosis. Atherosclerosis. 2015;242(1):357‐366. 26253795 10.1016/j.atherosclerosis.2015.07.035

[58] Geleijnse JM , Giltay EJ , Grobbee DE , Donders ART , Kok FJ . Blood pressure response to fish oil supplementation: metaregression analysis of randomized trials. J Hypertens. 2002;20(8):1493‐1499. 12172309 10.1097/00004872-200208000-00010

[59] Miller PE , Van Elswyk M , Alexander DD . Long‐chain omega‐3 fatty acids eicosapentaenoic acid and docosahexaenoic acid and blood pressure: a meta‐analysis of randomized controlled trials. Am J Hypertens. 2014;27(7):885‐896. 24610882 10.1093/ajh/hpu024 PMC4054797

[60] Mozaffarian D , Geelen A , Brouwer IA , Geleijnse JM , Zock PL , Katan MB . Effect of fish oil on heart rate in humans: a meta‐analysis of randomized controlled trials. Circulation. 2005;112(13):1945‐1952. 16172267 10.1161/CIRCULATIONAHA.105.556886

[61] von Schacky C , Fischer S , Weber PC . Long‐term effects of dietary marine omega‐3 fatty acids upon plasma and cellular lipids, platelet function, and eicosanoid formation in humans. J Clin Invest. 1985;76(4):1626‐1631. 2997286 10.1172/JCI112147 PMC424148

[62] Calder PC . Marine omega‐3 fatty acids and inflammatory processes: effects, mechanisms and clinical relevance. Biochim Biophys Acta. 2015;1851(4):469‐484. 25149823 10.1016/j.bbalip.2014.08.010

[63] Li K , Huang T , Zheng J , Wu K , Li D . Effect of marine‐derived n‐3 polyunsaturated fatty acids on C‐reactive protein, interleukin 6 and tumor necrosis factor alpha: a meta‐analysis. PLoS One. 2014;9(2):e88103. 24505395 10.1371/journal.pone.0088103 PMC3914936

[64] Thies F , Garry JMC , Yaqoob P , et al. Association of n‐3 polyunsaturated fatty acids with stability of atherosclerotic plaques: a randomised controlled trial. Lancet. 2003;361(9356):477‐485. 12583947 10.1016/S0140-6736(03)12468-3

[65] Konishi T , Sunaga D , Funayama N , et al. Eicosapentaenoic acid therapy is associated with decreased coronary plaque instability assessed using optical frequency domain imaging. Clin Cardiol. 2019;42(6):618‐628. 30993750 10.1002/clc.23185 PMC6553360

[66] Harris WS , Ginsberg HN , Arunakul N , et al. Safety and efficacy of Omacor in severe hypertriglyceridemia. J Cardiovasc Risk. 1997;4(5–6):385‐391. 9865671

[67] Serhan CN . Pro‐resolving lipid mediators are leads for resolution physiology. Nature. 2014;510(7503):92‐101. 24899309 10.1038/nature13479 PMC4263681

[68] Serhan CN , Chiang N , Dalli J . The resolution code of acute inflammation: novel pro‐resolving lipid mediators in resolution. Semin Immunol. 2015;27(3):200‐215. 25857211 10.1016/j.smim.2015.03.004 PMC4515371

[69] Siscovick DS , Barringer TA , Fretts AM , et al. Omega‐3 polyunsaturated fatty acid (fish oil) supplementation and the prevention of clinical cardiovascular disease: a science advisory from the American Heart Association. Circulation. 2017;135(15):e867‐e884. 28289069 10.1161/CIR.0000000000000482 PMC6903779

[70] Bhatt DL , Steg PG , Brinton EA , et al. Rationale and design of REDUCE‐IT: reduction of cardiovascular events with Icosapent ethyl‐intervention trial. Clin Cardiol. 2017;40(3):138‐148. 28294373 10.1002/clc.22692 PMC5396348

[71] HPS2‐THRIVE Collaborative Group , Haynes R , Jiang L , Hopewell JC , et al. HPS2‐THRIVE randomized placebo‐controlled trial in 25 673 high‐risk patients of ER niacin/laropiprant: trial design, pre‐specified muscle and liver outcomes, and reasons for stopping study treatment. Eur Heart J. 2013;34(17):1279‐1291. 23444397 10.1093/eurheartj/eht055 PMC3640201

[72] Guyton JR , Slee AE , Anderson T , et al. Relationship of lipoproteins to cardiovascular events: the AIM‐HIGH trial (Atherothrombosis intervention in metabolic syndrome with low HDL/high triglycerides and impact on Global Health outcomes). J Am Coll Cardiol. 2013;62(17):1580‐1584. 23916935 10.1016/j.jacc.2013.07.023 PMC3862446

[73] Araki E , Yamashita S , Arai H , et al. Effects of Pemafibrate, a novel selective PPARalpha modulator, on lipid and glucose metabolism in patients with type 2 diabetes and hypertriglyceridemia: a randomized, double‐blind, placebo‐controlled, phase 3 trial. Diabetes Care. 2018;41(3):538‐546. 29298800 10.2337/dc17-1589

[74] Leon H , Shibata MC , Sivakumaran S , Dorgan M , Chatterley T , Tsuyuki RT . Effect of fish oil on arrhythmias and mortality: systematic review. BMJ. 2008;337:a2931. 19106137 10.1136/bmj.a2931 PMC2612582

[75] Marik PE , Varon J . Omega‐3 dietary supplements and the risk of cardiovascular events: a systematic review. Clin Cardiol. 2009;32(7):365‐372. 19609891 10.1002/clc.20604 PMC6653319

[76] Rizos EC , Ntzani EE , Bika E , Kostapanos MS , Elisaf MS . Association between omega‐3 fatty acid supplementation and risk of major cardiovascular disease events: a systematic review and meta‐analysis. JAMA. 2012;308(10):1024‐1033. 22968891 10.1001/2012.jama.11374

[77] Alexander DD , Miller PE , Van Elswyk ME , et al. A meta‐analysis of randomized controlled trials and prospective cohort studies of eicosapentaenoic and docosahexaenoic long‐chain omega‐3 fatty acids and coronary heart disease risk. Mayo Clin Proc. 2017;92(1):15‐29. 28062061 10.1016/j.mayocp.2016.10.018

[78] Manson JE , Cook NR , Lee IM , et al. Marine n‐3 fatty acids and prevention of cardiovascular disease and cancer. N Engl J Med. 2018;380:23–32. 30415637 10.1056/NEJMoa1811403 PMC6392053

[79] Harris WS . Expert opinion: omega‐3 fatty acids and bleeding‐cause for concern? Am J Cardiol. 2007;99(6a):44c‐46c. 10.1016/j.amjcard.2006.11.021 17368278

[80] Wachira JK , Larson MK , Harris WS . N‐3 fatty acids affect haemostasis but do not increase the risk of bleeding: clinical observations and mechanistic insights. Br J Nutr. 2014;111(9):1652‐1662. 24472372 10.1017/S000711451300425X

[81] Itakura H , Yokoyama M , Matsuzaki M , et al. Relationships between plasma fatty acid composition and coronary artery disease. J Atheroscler Thromb. 2011;18(2):99‐107. 21099130 10.5551/jat.5876

[82] Nicholls SJ , Lincoff AM , Bash D , et al. Assessment of omega‐3 carboxylic acids in statin‐treated patients with high levels of triglycerides and low levels of high‐density lipoprotein cholesterol: rationale and design of the STRENGTH trial. Clin Cardiol. 2018;41(10):1281‐1288. 30125052 10.1002/clc.23055 PMC6489732

[83] Ballantyne CM , Bays HE , Kastelein JJ , et al. Efficacy and safety of eicosapentaenoic acid ethyl ester (AMR101) therapy in statin‐treated patients with persistent high triglycerides (from the ANCHOR study). Am J Cardiol. 2012;110(7):984‐992. 22819432 10.1016/j.amjcard.2012.05.031

[84] Bays HE , Ballantyne CM , Kastelein JJ , Isaacsohn JL , Braeckman RA , Soni PN . Eicosapentaenoic acid ethyl ester (AMR101) therapy in patients with very high triglyceride levels (from the multi‐center, placebo‐controlled, randomized, double‐blINd, 12‐week study with an open‐label extension [MARINE] trial). Am J Cardiol. 2011;108(5):682‐690. 21683321 10.1016/j.amjcard.2011.04.015

[85] Saito Y , Yokoyama M , Origasa H , et al. Effects of EPA on coronary artery disease in hypercholesterolemic patients with multiple risk factors: sub‐analysis of primary prevention cases from the Japan EPA lipid intervention study (JELIS). Atherosclerosis. 2008;200(1):135‐140. 18667204 10.1016/j.atherosclerosis.2008.06.003

[86] Bays HE , Braeckman RA , Ballantyne CM , et al. Icosapent ethyl, a pure EPA omega‐3 fatty acid: effects on lipoprotein particle concentration and size in patients with very high triglyceride levels (the MARINE study). J Clin Lipidol. 2012;6(6):565‐572. 23312052 10.1016/j.jacl.2012.07.001

[87] Ballantyne CM , Braeckman RA , Bays HE , et al. Effects of icosapent ethyl on lipoprotein particle concentration and size in statin‐treated patients with persistent high triglycerides (the ANCHOR study). J Clin Lipidol. 2015;9(3):377‐383. 26073397 10.1016/j.jacl.2014.11.009

